# Synthesis, Characterization and *in Vitro* Antitumor Activity of Platinum(II) Oxalato Complexes Involving 7-Azaindole Derivatives as Coligands

**DOI:** 10.3390/molecules190810832

**Published:** 2014-07-25

**Authors:** Pavel Štarha, Zdeněk Trávníček, Igor Popa, Zdeněk Dvořák

**Affiliations:** 1 Regional Centre of Advanced Technologies and Materials, Department of Inorganic Chemistry, Faculty of Science, Palacký University, 17. listopadu 12, CZ 77146 Olomouc, Czech Republic; E-Mails: pavel.starha@upol.cz (P.S.); igor.popa@upol.cz (I.P.); 2 Regional Centre of Advanced Technologies and Materials, Department of Cell Biology and Genetics, Faculty of Science, Palacký University, Šlechtitelů 11, CZ 78371 Olomouc, Czech Republic; E-Mail: zdenek.dvorak@upol.cz

**Keywords:** platinum(II) complexes, oxalato complexes, 7-azaindole derivatives, multinuclear NMR, antitumor activity

## Abstract

The platinum(II) oxalato complexes [Pt(ox)(*n*aza)_2_] (**1**–**3**) were synthesized and characterized by elemental analysis (C, H, N), multinuclear NMR spectroscopy (^1^H, ^13^C, ^15^N, ^195^Pt) and electrospray ionization mass spectrometry (ESI-MS); *n*aza = 4-chloro-7-azaindole (*4Cl*aza; **1**), 3-bromo-7-azaindole (*3Br*aza; **2**) or 4-bromo-7-azaindole (*4Br*aza; **3**). The prepared substances were screened for their *in vitro* antitumor activity on the osteosarcoma (HOS) and breast adenocarcinoma (MCF7) human cancer cell lines, where **2** showed moderate antitumor effect (IC_50_ = 27.5 μM, and 18.3 μM, respectively). The complex **2** was further tested on a panel of six others human cancer cell lines, including the malignant melanoma (G361), cervix carcinoma (HeLa), ovarian carcinoma (A2780), *cisplatin*-resistant ovarian carcinoma (A2780R), lung carcinoma (A549) and prostate adenocarcinoma (LNCaP). This substance was found to be moderate antitumor effective against G361 (IC_50_ = 17.3 μM), HeLa (IC_50_ = 31.8 μM) and A2780 (IC_50_ = 19.2 μM) cell lines. The complex **2** was also studied by NMR for its solution stability and by ESI-MS experiments for its ability to interact with biomolecules, such as cysteine, glutathione or guanosine 5'-monophosphate.

## 1. Introduction

Platinum carboxylates represent a notable group of transition metal complexes, which have been used for the treatment of various types of cancer for many years [1,2,3]. Various carboxylate anions involved in the structures of the clinically used or studied platinum-based metallotherapeutics can be mentioned, particularly the cyclobutane-1,1-dicarboxylate dianion (involved in carboplatin), glycolate dianion (involved in nedaplatin), lactate dianion (involved in lobaplatin), malonate dianion (involved in heptaplatin), acetate anion (involved in satraplatin) or neodecanoate anion (involved in aroplatin) [4,5,6,7,8,9]. One of the carboxylate-based leaving groups is the oxalate dianion involved in the well-known substance oxaliplatin clinically used mainly for the treatment of colorectal tumours [10]. In other words, the platinum(II) oxalato complexes are, thanks to the mentioned oxaliplatin, biologically relevant and worth studying group of compounds.

Search for novel antitumor active platinum complexes in terms of novel carrier (*i.e.*, *N*-donor ligands) and leaving (*i.e.*, carboxylates) ligands is one of the crucial challenge of modern bioinorganic chemistry [11,12,13], although those involved in clinically used drugs are still substantial part of this research, as exemplified by the NH_3_ carrier ligands involved in original platinum-based drug cisplatin, as well as in currently studied picoplatin [14]. As for the biologically effective platinum oxalato complexes, it is quite interesting that although these complexes can be considered, thanks to clinically used anticancer drug oxaliplatin [15], as biologically perspective group of compounds [16], not many papers dealing with such compounds in connection with their biological effect have been reported in last five years [17,18,19,20,21,22,23,24]. Our research group reported the platinum(II) oxalato complexes involving variously substituted *N*6-benzyladenine derivatives [17,18,19], whose the most effective representatives showed IC_50_(HOS) = 3.6 µM ([Pt(ox)(L_1_)]; L_1_=2-chloro-*N*6-(2-methoxybenzyl)-9-isopropyladenine) and IC_50_(MCF7) = 3.6 µM ([Pt(ox)(L_2_)]; L_2_=2-chloro-*N*6-(2,4-dimethoxybenzyl)-9-isopropyladenine). Utku *et al.* described the platinum(II) oxalato complexes with 2-phenylbenzimidazole ligand and their antibacterial and antifungal activity [20]. The work of Silva *et al.* dealt with the [Pt(ox)(L_3_)] (L_3_ symbolizes a long-chain aliphatic diamine) and their cytotoxic effect against A549, B16-F1, B16-F10 and MDA-MB-231 cancer cells and BHK-21 and CHO non-cancer cells. The obtained results proved four reported oxalato complexes as less active against the named cancer cells (IC_50_(A549) = 12.6–60.6 µM, IC_50_(B16-F1) = 14.5–21.9 µM, IC_50_(B16-F10) = 16.6–38.0 µM, IC_50_(MDA-MB-231) = 16.6–25.1 µM) as compared with cisplatin (IC_50_(A549) = 2.7 µM, IC_50_(B16-F1) = 3.5 µM, IC_50_(B16-F10) = 4.2 µM, IC_50_(MDA-MB-231) = 1.4 µM) [21]. Other platinum(II) oxalato complexes involved 1,2-diaminocyclohexane with variously monoalkyl-substituted nitrogen atom (L_4_) [22]. These substances were studied for their *in vitro* cytotoxicity against HepG-2, MCF7, A549 and HCT-116 human cancer cell lines. The antitumor effect of the most effective complex, expressed as IC_50_ = 3.7, 8.8, 18.4, and 2.1 µM, respectively, against the mentioned cells, exceed both cisplatin and oxaliplatin used as standards in this study. The biological perspective of the oxalate anion, used as *O*-donor ligand in the biologically active transition metal complexes, can be also demonstrated on the palladium(II) oxalato complexes with *N*6-benzyladenin-based *N*-donor ligands, which in many cases showed noticeable antitumor activity against human cancer cell lines in many cases [19,25,26].

**Scheme 1:**
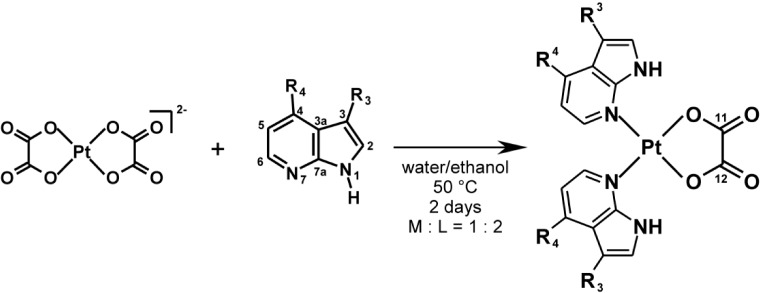
The synthesis and schematic representation of the prepared [Pt(ox)(*n*aza)_2_] complexes (**1**–**3**) given with the atom numbering scheme of the used 7-azaindole derivatives.

The herein described platinum(II) oxalato complexes with variously substituted 7-azaindole halogeno-derivatives (**1**–**3**; Scheme 1), namely 4-chloro-7-azaindole (*4Cl*aza; the complex **1**), 3-bromo-7-azaindole (*3Br*aza; the complex **2**) and 4-bromo-7-azaindole (*4Br*aza; the complex **3**), follow recently reported analogues involving differently substituted 7-azaindoles (3-chloro-7-azaindole, *3Cl*aza; 3-iodo-7-azaindole, *3I*aza; 5-bromo-7-azaindole, *5Br*aza) [24]. Although the mentioned recently reported platinum(II) oxalato complexes with *3Cl*aza, *3I*aza and *5Br*aza *N*-donor ligands did not show any biological effect on osteosarcoma HOS, breast carcinoma MCF7 and prostate carcinoma LNCaP human cancer cell lines (these complexes were poorly soluble in the medium used), we decided to study, whether substitution variability of the 7-azaindole moiety within the platinum(II) oxalato complexes may led to the formation of antitumor active compounds.

R_3_=H and R_4_=Cl for *4Cl*aza and [Pt(ox)(*4Cl*aza)_2_] (**1**); R_3_=Br and R_4_=H for *3Br*aza and [Pt(ox)(*3Br*aza)_2_] (**2**) and R_3_=H and R_4_=Br for *4Br*aza and [Pt(ox)(*4Br*aza)_2_] (**3**).

## 2. Results and Discussion

### 2.1. General Properties

The platinum(II) oxalato complexes [Pt(ox)(*n*aza)_2_] (**1**–**3**; Scheme 1) were prepared by a one-step synthetic procedure using the bis(oxalato)platinate(II) salt as a starting compound [17]. The composition of the products was proved by the results of elemental analysis. The electrospray ionization mass spectra obtained in the positive mode (ESI+) of the studied complexes contained the molecular peaks detected at 589.2 *m/z* (calc. 589.0; 5%; {[Pt(ox)(*4Cl*aza)_2_]+H}^+^ for **1**), 678.9 *m/z* (calc. 678.9; 40%; {[Pt(ox)(*3Br*aza)_2_]+H}^+^ for **2**) and 679.0 *m/z* (calc. 678.9; 25%; {[Pt(ox)(*4Br*aza)_2_]+H}^+^for **3**), their adducts with different cations, namely 627.1 *m/z* (calc. 626.9; 30%; {[Pt(ox)(*4Cl*aza)_2_]+K}^+^ for **1**), 611.1 *m/z* (calc. 611.0; 50%; {[Pt(ox)(*4Cl*aza)_2_]+Na}^+^ for **1**), 716.9 *m/z* (calc. 716.8; 100%; {[Pt(ox)(*3Br*aza)_2_]+K}^+^ for **2**), 701.0 *m/z* (calc. 700.9; 15%; {[Pt(ox)(*3Br*aza)_2_]+Na}^+^ for **2**), 716.9 *m/z* (calc. 716.8; 100%; {[Pt(ox)(*4Br*aza)_2_]+K}^+^ for **3**) and 701.1 *m/z* (calc. 700.9; 55%; {[Pt(ox)(*4Br*aza)_2_]+Na}^+^ for **3**). The peak of the released *N*-donor ligand was found at 153.1 *m/z* (calc. 153.0; 5%; {*4Cl*aza+H}^+^ for **1**), 197.0 *m/z* (calc. 197.0; 20%; {*3Br*aza+H}^+^ for **2**) and 197.1 *m/z* (calc. 197.0; 15%; {*4Br*aza+H}^+^ for **3**). The pseudomolecular peaks of the {[Pt(ox)(*n*aza)_2_]−H}^–^ species were detected by means of ESI− (electrospray ionization in the negative mode) mass spectrometry at 585.9 *m/z* (calc. 586.0; 60%; {[Pt(ox)(*4Cl*aza)_2_]−H}^−^ for **1**) and 676.0 *m/z* (isomeric complexes **2** and **3**; calc. 675.9; 100%; {[Pt(ox)(*3Br*aza)_2_]−H}^–^ for **2**, and: calc. 675.9; 85%; {[Pt(ox)(*4Br*aza)_2_]–H}^−^ for **3**). The ESI- mass spectra of the studied compounds also contain the peaks whose mass correspond to the {[Pt(ox)(*n*aza)]−H}^−^ fragment (434.0 *m/z*, calc. 434.0; 30%; {[Pt(ox)(*4Cl*aza)]−H}^−^ for **1**; 478.1 *m/z*, calc. 477.9; 30%; {[Pt(ox)(*3Br*aza)]−H}^−^ for **2**; 478.1 *m/z*, calc. 477.9; 65%; {[Pt(ox)(*4Br*aza)]−H}^−^ for **3**) and {*n*aza−H}^−^ ligand (151.0 *m/z*, calc. 151.0; 10%; {*4Cl*aza−H}^−^ for **1**; 195.1 *m/z*, calc. 195.0; 15%; {*3Br*aza−H}^−^ for **3**; 195.1 *m/z*, calc. 195.0; 20%; {*4Br*aza−H}^−^ for **3**).

The complexes **1**–**3**, as well as the starting compounds *n*aza and K_2_[Pt(ox)_2_]∙2H_2_O, were studied by means of multinuclear and 2D NMR spectroscopy. All the ^1^H, ^13^C and ^15^N signals of free *n*aza molecules were unambiguously detected in the spectra of corresponding platinum(II) complexes and assigned by means of the below-mentioned 2D NMR experiments. The significantly different ^15^N-NMR coordination shift values of the N1 (|Δδ| = 2.5–3.4 ppm) and N7 (|Δδ| = 114.0–115.1 ppm) atoms clearly proved the coordination of *n*aza molecules through the N7 atoms. The ^13^C-NMR chemical shifts of the C11 and C12 atoms of the oxalate dianion (detected at 165.9–166.0 ppm) and the ^195^Pt chemical shifts, which equal −1770.1 (**1**), −1783.5 (**2**) and −1772.5 (**3**), correlate well with the values of the formerly reported platinum(II) oxalato complexes with 7-azaindole [23] or its derivatives [24].

### 2.2. NMR and ESI-MS Stability and Interaction Studies

**Figure 1:**
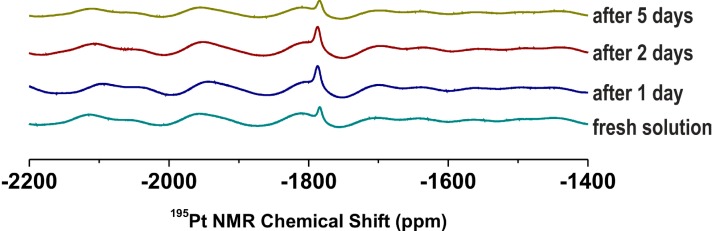
Time dependent ^195^Pt NMR spectra on the representative complex **2** dissolved in DMF-*d_7_*/H_2_O mixture (9:1 *v/v*) showing on the stability of the complex.

^1^H and ^195^Pt NMR spectroscopy (solution of **2** in DMF-*d_7_* and DMF-*d_7_*/H_2_O mixture, 9:1 *v/v*) and electrospray ionization mass spectrometry (ESI-MS; solution of **2** in methanol and water/methanol mixture, 1:1 *v/v*) (the presence of the organic solvents ensured the solubility of the studied complex, because carrying out of the experiments in water was prevented by limited solubility of the mentioned complex in water) were used to investigate the behaviour of the representative complex **2** in the mentioned organic or water-containing solvents. As it is generally accepted for the antitumor active platinum(II) complexes, hydrolysis is a crucial step within the mechanism of action, which lead to the replacement of leaving groups (*i.e.*, the oxalato ligands) and formation of the activated and more reactive aqua- and/or hydroxidoplatinum(II) species, probably with formulas *cis*-[Pt(H_2_O)_2_(*3Br*aza)_2_]^2+^ and/or *cis*-[Pt(OH)_2_(*3Br*aza)_2_] in our case [1,2,27,28]. The hydrolysis of the platinum(II) oxalate complexes should be connected with opening of the PtO_2_C_2_ ring and/or substitution of the oxalate dianion by two H_2_O or OH^−^ species as ligands, both resulting in the change of inner coordination sphere (resulting in new peaks in mass spectra) and electron density within the initial complex, which is known to provide different ^1^H and ^195^Pt NMR chemical shifts [27,28]. Since we did not observe any new signals in both the ^1^H and ^195^Pt NMR spectra (Figure 1), it can be concluded that the complex **2** is stable and do not undergo any changes within the structure during 5 days in DMF-*d_7_* as well as in the DMF-*d_7_*/H_2_O mixture. Similarly it was found that the complex is stable and did not show any change in the composition from the mass spectrometry point of view, because its mass spectra (methanol solutions) recorded after 12 h did not contain any novel peaks as compared with the spectra obtained on the fresh solution of **2**.

**Figure 2:**
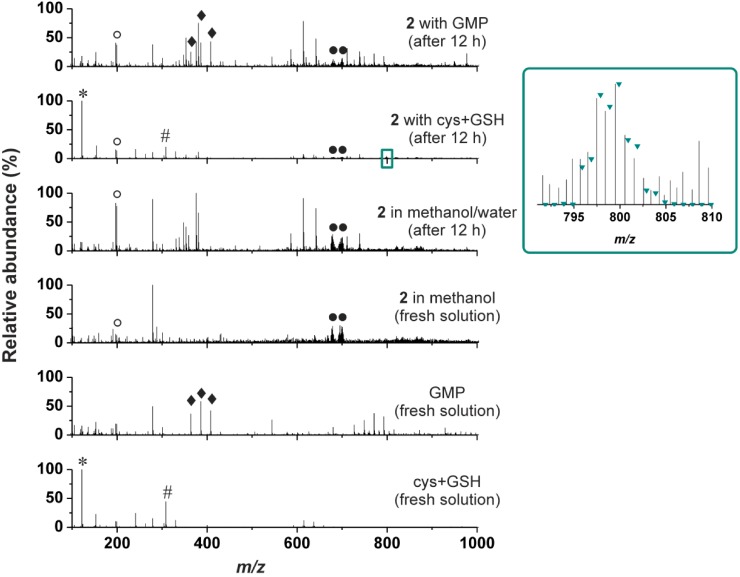
The ESI+ mass spectra (recorded on fresh solutions and after 12 h) for the stability in methanol/water mixture, and for the interaction of the representative complex **2** with the mixture of cysteine and reduced glutathione (cys+GSH), and guanosine 5'-monophosphate (GMP), given together with the spectra of the fresh solutions of the individual reactants, *i.e.*, the complex **2**, cys+GSH mixture and GMP.

In the case of water/methanol mixture solution of **2**, we detected several new peaks in the mass spectra in comparison with the spectra of **2** dissolved in pure methanol, but their isotopic distribution did not correspond to that of platinum-containing species (Figure 2). In other words, no new platinum-containing species was found in the mass spectra recorded on the water/methanol solution of **2**, which, as in the case of NMR, proved that no hydrolytic processes proceeded under the experimental condition used. Thus it can be said that we did not get any evidence of the hydrolysis usually involved within the mechanism of action of the cytotoxic active platinum(II) complexes including the clinically used platinum(II) oxalate complex oxaliplatin [1,2,29]. On the other hand, it is known that the oxaliplatin hydrolysis in water (leading to diaqua-species) and under *in vivo* conditions has different course, since the latter one provides the carbonato or phosphato adducts (instead of the above mentioned diaqua ones), which consequently enhance the reactivity of such species towards nucleophiles including nucleobases [30,31,32]. It means that although we did not observed any processes usually associated with the action of cytotoxic platinum(II) complexes (see Section Section 2.3. for the *in vitro* cytotoxicity of **1**–**3**) under experimental conditions, the cytotoxic action itself is not excluded with respect to different conditions in the cells (cytosol involving various ions) as compared with those used in the herein discussed NMR and ESI-MS experiments.

ESI-MS was also used to study the ability of **2** to interact with sulphur-containing biomolecules (cysteine (cys) and reduced glutathione (GSH)) or guanosine 5'-monophosphate (GMP) in water/methanol mixture (1:1 *v/v*) (again, the presence of the organic solvent ensured the solubility of the studied complex). It is well-known that ability of the cytotoxic platinum(II) complexes to interact with the intracellular sulphur-containing compounds correlates with their activity as well as with the resistance of the respective tumours in terms of inactivation of the platinum(II) species and their removing from the cell [1,29]. With respect to this phenomena, ability of the studied platinum(II) complexes to interact with the sulphur-containing biomolecules should be investigated by relevant techniques. In the case of this work, we studied an interaction of **2** with the mixture of cys and GSH in water/methanol mixture. We did not observe any adducts of **2** (or its fragments formed during ionization) with cysteine or reduced glutathione assignable to the species formed by their interaction (Figure 2), which corresponds to the above-described reluctance of **2** to undergo hydrolysis. The only exception from this statement is very weak peak of the {[Pt(cys)(ox)(*3Br*aza)_2_]+H}^+^ species (Figure 2, inset) detected in the ESI+ spectra of the studied complex **2** at 799.5 *m/z* (calcd. 799.9 *m/z*), which most probably contains a ring-opened product of the interaction with a monodentate bound oxalate dianion.

* stands for {cys+H}^+^, ♯ for {GSH+H}^+^,♦ for {H_2_GMP+H}^+^, {HNaGMP+H}^+^ and {Na_2_GMP+H}^+^, ○ for {*3Br*aza+H}^+^ and ● for {[Pt(ox)(*3Br*aza)_2_]+H}^+^ and {[Pt(ox)(*3Br*aza)_2_]+Na}^+^; *Inset*: Part of the ESI+ mass spectrum of the mixture of the complex **2** with cys+GSH (recorded 12 h after the preparation) showing the peak of the {[Pt(cys)(ox)(*3Br*aza)_2_]+H}^+^ species, together with the simulated isotopic distribution labeled as the green triangles.

The mechanism of action itself of the clinically used antitumor active platinum(II) complexes is based on the covalent binding of activated platinum(II) species to the nuclear DNA molecule of the tumour cells [33], which is also expected for most of the platinum(II) complexes having cytotoxic effect. A simple model to study the ability of the platinum(II) complexes to bind DNA molecule is based on binding reactions with various nucleobase-based compounds such as GMP employed in this work. However, we have to state that we did not detect any species whose mass and isotopic distribution would correspond with those of adduct of the studied complex, its fragments or its hydrolysis products with GMP (Figure 2).

### 2.3. In Vitro Cytotoxicity

**Figure 3:**
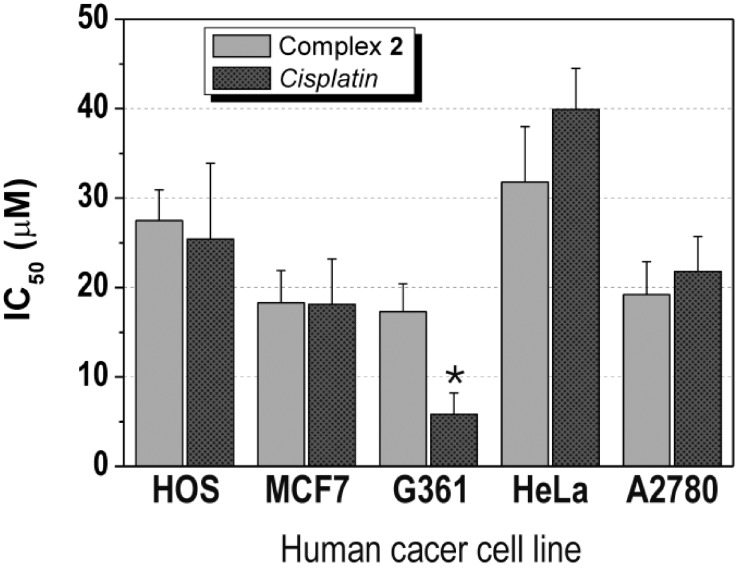
The *in vitro* antitumor activity of the complex **2** and cisplatin (CDDP) on osteosarcoma (HOS), breast adenocarcinoma (MCF7), malignant melanoma (G361), cervix carcinoma (HeLa) and ovarian carcinoma (A2780) human cancer cell lines.

The complexes **1**–**3** were screened for their *in vitro* antitumor activity against two types of the human cancer cell lines - osteosarcoma (HOS) and breast adenocarcinoma (MCF7) (Table 1). In the case of **1** and **3**, the testing was limited by low solubility of the compounds, which can be expressed as <1.0 μM. Interestingly, the solubility of **2** was much higher (>50.0 μM) in the medium used. This compound showed the moderate *in vitro* anticancer activity, concretely 27.5 ± 3.4 μM against HOS and 18.3 ± 3.6 μM against MCF7 cells, which is comparable effect with that of cisplatin (IC_50_(HOS) = 25.4 ± 8.5 μM, IC_50_(MCF7) = 18.1 ± 5.1 μM) (Table 1, Figure 3). It has to be mentioned, that we also tried to compare the results of **2** with another platinum-based drug, oxaliplatin, which involve the same living group in its structure as the studied complexes **1**–**3**. However, such comparison is limited by the fact that oxaliplatin did not show any cytotoxic effect on both cell lines up to the 50.0 μM concentration (IC_50_(HOS) > 50.0 μM, IC_50_(MCF7) > 50.0 μM). Nevertheless, it can be stated that the complex **2** exceeded the *in vitro* antitumor activity of oxaliplatin on HOS and MCF7 human cancer cell lines.

**Table 1:** The results of the *in vitro* antitumor activity of the studied platinum(II) oxalato complexes (**1**–**3**), cisplatin (CDDP) and oxaliplatin (OXA) againstosteosarcoma (HOS), breast adenocarcinoma (MCF7), malignant melanoma (G361), cervix carcinoma (HeLa), ovarian carcinoma (A2780), cisplatin-resistant ovarian carcinoma (A2780R), lung carcinoma (A549) and prostate carcinoma (LNCaP) human cancer cell lines, as obtained by an MTT assay on the cells exposed to the compounds for 24 h.

	HOS	MCF7	G361	HeLa	A2780	A2780R	A549	LNCaP
**1**	>1.0	>1.0	nt	nt	nt	nt	nt	nt
**2**	27.5 ± 3.4	18.3 ± 3.6	17.3 ± 3.1	31.8 ± 6.2	19.2 ± 3.7	>50.0	>50.0	>50.0
**3**	>1.0	>1.0	nt	nt	nt	nt	nt	nt
CDDP	25.4 ± 8.5	18.1 ± 5.1	5.8 ± 2.4	39.9 ± 4.6	21.8 ± 3.9	32.0 ± 9.6	>50.0	3.8 ± 1.5
OXA	>50.0	>50.0	>50.0	>50.0	>50.0	>50.0	>50.0	>50.0

With respect to the results obtained on HOS and MCF7, the complex **2** was tested against next six human cancer cell lines, namely malignant melanoma (G361), cervix carcinoma (HeLa), ovarian carcinoma (A2780), cisplatin-resistant ovarian carcinoma (A2780R), lung carcinoma (A549) and prostate adenocarcinoma (LNCaP). It has been observed that its *in vitro* antitumor activity equals IC_50_ = 17.3 ± 3.1 μM (G361; 5.8 ± 2.4 μM for cisplatin, >50.0 μM for oxaliplatin), IC_50_ = 31.8 ± 6.2 μM (HeLa; 39.9 ± 4.6 μM for cisplatin, >50.0 μM for oxaliplatin), IC_50_ = 19.2 ± 3.7 μM (A2780; 21.8 ± 3.9 μM for cisplatin, >50.0 μM for oxaliplatin) and IC_50_ > 50.0 μM (A2780R, A549 and LNCaP; 32.0 ± 9.6, >50.0 and 3.8 ± 1.5 μM for cisplatin, respectively, >50.0 μM for oxaliplatin) cell lines (Table 1, Figure 3). The antitumor activity of **2** against G361, HeLa, A2780, A2780R, A549 and LNCaP can be evaluated as moderate and slightly higher on A2780 and HeLa in comparison with clinically used platinum-based therapeutic cisplatin and on G361, A2780 and HeLa as compared with another platinum-based drug oxaliplatin.

As it is mentioned above, **1**–**3** follow recently reported [24] analogous oxalato complexes involving different types of 7-azaindole halogeno-derivatives, concretely 3-chloro-7-azaindole (*3Cl*aza), 3-iodo-7-azaindole (*3I*aza) and 5-bromo-7-azaindole (*5Br*aza), which, similarly to **1** and **3**, did not show any effect against both HOS and MCF7 cell lines up to the concentration of 10.0, 25.0, and 0.5 μM, respectively. Concerning all six platinum(II) oxalato complexes with different 7-azaindole derivatives together, it can be said that the biological activity, in terms of bioavailability, is strongly affected by the position of halogeno-substituent of the 7-azaindole moiety, because the solubility of the complexes with *3Cl*aza, *3Br*aza and *3I*aza (10.0–50.0 μM) is significantly higher than that of the complexes involving the 7-azaindole derivatives substituted in the position 4 (*4Cl*aza and *4Br*aza) or 5 (*5Br*aza), whose solubility did not exceed 1.0 μM in the medium used.

Asterisk (*) symbolizes significant difference (*p* < 0.05) in *in vitro* antitumour activity of **2** as compared to *cisplatin*; nt = not tested.

## 3. Experimental Section

### 3.1. Materials and Methods

Potassium tetrachloridoplatinate(II) (K_2_[PtCl_4_]), potassium oxalate monohydrate (K_2_(ox)∙H_2_O), 4-chloro-7-azaindole (*4Cl*aza), 3-bromo-7-azaindole (*3Br*aza), 4-bromo-7-azaindole (*4Br*aza), *cisplatin*, *oxaliplatin*, cysteine (cys), reduced glutathione (GSH), guanosine 5'-monophosphate disodium salt (GMP) and solvents were purchased from Sigma-Aldrich Co. (Prague, Czech Republic) and Acros Organics Co. (Pardubice, Czech Republic) and used as received.

*General methods*: Elemental analysis was performed on a Flash 2000 CHNS Elemental Analyzer (Thermo Scientific, Waltham, MA, USA).

*Electrospray ionization mass spectrometry (ESI-MS)*: Mass spectra were obtained on fresh methanol solutions and after 2 h and 12 h by an LCQ Fleet ion trap mass spectrometer using both the positive (ESI+) negative (ESI−) mode electrospray ionization technique (Thermo Scientific, QualBrowser software, version 2.0.7, Thermo Fischer Scientific, Waltham, MA, USA). The 10 µM (final concentration) solution of **2** in methanol was mixed together with the same volume of water (hydrolysis studies; presence of methanol ensured the solubility of the studied complex, because carrying out of the experiments in water was prevented by limited solubility of the mentioned complex in water), water solutions of the mixture of GSH (6 µM) and cys (260 µM) or water solution of GMP. 20 µL of the mixtures was analysed by means of flow injection analysis/mass spectrometry (FIA/ESI-MS) in both the positive and negative ionization modes 0 h, 2 h and 12 h after the preparation.

*NMR spectroscopy*: ^1^H, ^13^C and ^195^Pt NMR spectra and two dimensional correlation ^1^H-^1^H gs-COSY, ^1^H-^13^C gs-HMQC, ^1^H-^13^C gs-HMBC and ^1^H-^15^N gs-HMBC experiments (DMF-*d_7_* solutions) were performed at 300 K on a Varian 400 device (Santa Clara, CA, USA) at 400.00 MHz (^1^H), 100.58 MHz (^13^C), 86.00 MHz (^195^Pt) and 40.53 MHz (^15^N); gs = gradient selected, COSY = correlation spectroscopy, HMQC = heteronuclear multiple quantum coherence, HMBC = heteronuclear multiple bond coherence. ^1^H and ^13^C spectra were adjusted against SiMe_4_, while ^195^Pt spectra were calibrated against K_2_[PtCl_6_] in D_2_O found at 0 ppm. ^1^H-^15^N gs-HMBC experiments were obtained at natural abundance and calibrated against the residual signals of DMF (8.03 ppm for ^1^H, 104.7 ppm for ^15^N). The splitting of proton resonances in the reported ^1^H-NMR spectra is defined as s = singlet, d = doublet, t = triplet, br = broad band, m = multiplet. The coordination shift (Δδ; ppm) is calculated as Δδ = δ_complex_ − δ_ligand_. The stability studies of the DMF-*d_7_* and DMF-*d_7_*/H_2_O (9:1 *v/v*) solutions of **2** was carried out by means of ^1^H and ^195^Pt NMR after 1, 2, 3, 4 and 5 days of standing at laboratory temperature. 

### 3.2. Synthesis of Complexes **1**–**3**

A solution of 1.0 mmol of *4Cl*aza (for **1**), *3Br*aza (for **2**) or *4Br*aza (for **3**) in 10 mL of hot (50 °C) ethanol was slowly poured into the solution of K_2_[Pt(ox)_2_]∙2H_2_O (0.5 mmol) in 10 mL of hot (50 °C) distilled water. The reaction mixtures were stirred at 50 °C for two days. The products, which formed, were filtered off, washed (5 mL of distilled water and 5 mL of ethanol) and dried at 40 °C (Figure 1). The described syntheses followed a procedure reported in our recent works for analogous complexes with different 7-azaindoles [23,24].

*Bis(4-chloro-7-azaindole)-κN7}(oxalato-κ^2^O,O’)platinum(II)* (**1**, C_16_H_10_N_4_Cl_2_O_4_Pt) Light grey solid; yield 80%; ^1^H-NMR (400.0 MHz, DMF-*d_7_*): *δ/Δδ* = 13.41/1.34 (br, NH-1), 8.71/0.49 (d, *J* = 6.4, CH-6), 7.98/0.30 (d, *J* = 3.6, CH-2), 7.37/0.17 (d, *J* = 6.3, CH-5), 6.79/0.21 (d, *J* = 3.6, CH-3) ppm. ^13^C-NMR (100.6 MHz, DMF-*d_7_*): *δ/Δδ* = 165.9 (C-11,12), 148.2/−1.6 (C-7a), 146.6/3.0 (CH-6), 138.4/3.8 (C-4), 129.3/1.9 (CH-2), 122.2/3.1 (C-3a), 117.5/1.9 (CH-5), 100.5/2.2 (CH-3) ppm. ^15^N-NMR (40.5 MHZ, DMF-*d_7_*): *δ/Δδ* = 145.6/3.4 (NH-1), 154.6/−114.6 (N-7) ppm. ^195^Pt NMR (86.0 MHz, DMF-*d_7_*): *δ* = −1770.1 ppm. ESI MS (30 ev): *m/z* = 611.1 (M+Na), 585.9 (M−H), 434.0 (M−*4Cl*aza−H), 153.1 (*4Cl*aza+H), 151.0 (*4Cl*aza−H). Anal. Calc.: C, 32.7%; H, 1.7%; N, 9.5%. Found: C, 32.8%; H, 1.6%; N, 9.6%.

*Bis(3-bromo-7-azaindole)-κN7}(oxalato-κ^2^O,O’)platinum(II)* (**2**, C_16_H_10_N_4_Br_2_O_4_Pt) Light grey solid; yield 75%; ^1^H-NMR (400.0 MHz, DMF-*d_7_*): *δ/Δδ* = 13.49/1.32 (br, NH-1), 8.82/0.48 (d, *J* = 5.7, CH-6), 8.11/0.23 (d, *J* = 8.0, CH-4), 8.09/0.31 (s, CH-2), 7.31/0.10 (m, CH-5) ppm. ^13^C-NMR (100.6 MHz, DMF-*d_7_*): *δ/Δδ* = 166.0 (C-11,12), 147.5/3.2 (CH-6), 147.0/−0.9 (C-7a), 130.6/4.0 (CH-4), 128.0/2.1 (CH-2), 122.6/3.3 (C-3a), 118.0/1.4 (CH-5), 89.3/1.6 (C-3) ppm. ^15^N-NMR (40.5 MHZ, DMF-*d_7_*): *δ/Δδ* = 143.5/2.5 (NH-1), 159.4/−115.1 (N-7). ^195^Pt NMR (86.0 MHz, DMF-*d_7_*): *δ* = −1783.5 ppm. ESI MS (30 ev): *m/z* = 678.9 (M+H), 676.0 (M−H), 478.1 (M−*3Br*aza−H), 197.1 (*3Br*aza+H), 195.1 (*3Br*aza–H). Anal. Calc.: C, 28.4%; H, 1.5%; N, 8.3%. Found: C, 28.4%; H, 1.5%; N, 8.3%.

*Bis(4-bromo-7-azaindole)-κN7}(oxalato-κ^2^O,O’)platinum(II)* (**3**, C_16_H_10_N_4_Br_2_O_4_Pt) Light grey solid; yield 80%; ^1^H-NMR (400.0 MHz, DMF-*d_7_*): *δ/Δδ* = 13.40/1.31 (br, NH-1), 8.60/0.47 (d, *J* = 6.3, CH-6), 8.00/0.30 (d, *J* = 3.5, CH-2), 7.51/0.16 (d, *J* = 6.2, CH-5), 6.72/0.21 (d, *J* = 3.5, CH-3) ppm. ^13^C-NMR (100.6 MHz, DMF-*d_7_*): *δ/Δδ* = 165.9 (C-11,12), 147.3/−1.6 (C-7a), 146.3/2.9 (CH-6), 129.4/1.9 (CH-2), 128.0/4.1 (C-3a), 124.6/3.2 (C-4), 120.6/1.8 (CH-5), 102.1/2.2 (CH-3) ppm. ^15^N-NMR (40.5 MHZ, DMF-*d_7_*): *δ/Δδ* = 145.5/3.1 (NH-1), 155.4/−114.0 (N-7). ^195^Pt NMR (86.0 MHz, DMF-*d_7_*): *δ* = −1772.5 ppm. ESI MS (30 ev): *m/z* = 679.0 (M+H), 676.0 (M−H), 478.1 (M−*4Br*aza−H), 197.1 (*4Br*aza+H), 195.1 (*4Br*aza−H). Anal. Calc.: C, 28.4%; H, 1.5%; N, 8.3%. Found: C, 28.3%; H, 1.5%; N, 8.4%.

### 3.3. In Vitro Cytotoxicity Testing

Breast adenocarcinoma (MCF7; ECACC No. 86012803), osteosarcoma (HOS; ECACC No. 87070202), malignant melanoma (G361; ECACC No. 88030401), cervix epitheloid carcinoma (HeLa; ECACC No. 93021013), A2780 ovarian carcinoma (ECACC No. 93112519), A2780R cisplatin-resistant ovarian carcinoma (ECACC No.93112517), lung carcinoma (A549; ECACC No.86012804) and prostate adenocarcinoma (LNCaP; ECACC No. 89110211) cancer cell lines were purchased from European Collection of Cell Cultures (ECACC; Prague, Czech Republic).

*In vitro* cytotoxicity was determined by an MTT assay against MCF7 (the complexes **1**–**3**), HOS (**1**–**3**), G361 (**2**), HeLa (**2**), A2780 (**2**), A2780R (**2**), A549 (**2**) and LNCaP (**2**) human cancer cell lines. The cells were maintained in a humidified incubator (37 °C, 5% CO_2_). The cells were treated with **1**–**3** or standards (cisplatin, oxaliplatin) at the 0.01, 0.1, 1.0, 5.0, 25.0 and 50 μM concentrations for 24 h, using multi-well culture plates of 96 wells. In parallel, the cells were treated with vehicle (DMF; 0.1%, *v*/*v*) and Triton X-100 (1%, *v*/*v*) to assess the minimal (*i.e.*, positive control) and maximal (*i.e.*, negative control) cell damage, respectively. The MTT assay was measured spectrophotometrically at 540 nm (TECAN, Schoeller Instruments LLC). The data were expressed as the percentage of viability, when 100% and 0% represent the treatments with DMF and Triton X-100, respectively. The cytotoxicity data from the cancer cell lines were acquired from three independent experiments (conducted in triplicate) using cells from different passages. The IC_50_ values (µM) were calculated from viability curves. The results are presented as arithmetic mean ± SD.

The significance of the differences between the results was assessed by the ANOVA analysis, followed by Tukey’s post-hoc test for multiple comparisons, with *p* < 0.05 considered to be significant (QC Expert 3.2, Statistical software, TriloByte Ltd., Pardubice, Czech Republic).

## 4. Conclusions

This work describes three new platinum(II) oxalato complexes [Pt(ox)(*n*aza)_2_] (**1**–**3**) and evaluates their *in vitro* cytotoxicity on the selected human cancer cell lines. The testing revealed the complex **2** (involving *3Br*aza) as *in vitro* antitumor active against HOS, MCF7, G361, HeLa and A2780 with IC_50_° 17–32 µM. Because the complex **2** differs from **1** (involving *3Cl*aza) and **3** (involving *4Br*aza) in the nature or position of the 7-azaindole moiety substituent, it can be concluded that *3Br*aza represents a very perspective *N*-donor ligand, which could be used as a carrier ligand involved into platinum(II) complexes with different leaving group than oxalato one.
